# Patterns of physical activity and health-related quality of life amongst patients with multimorbidity in a multi-ethnic Asian population

**DOI:** 10.1186/s12889-019-7941-4

**Published:** 2019-12-02

**Authors:** Mythily Subramaniam, Yunjue Zhang, Jue Hua Lau, Janhavi Ajit Vaingankar, Edimansyah Abdin, Siow Ann Chong, Eng Sing Lee

**Affiliations:** 1 Research Division Institute of Mental Health, Buangkok Green Medical Park, 10 Buangkok View, Singapore, 539747 Singapore; 2 National Healthcare Group Polyclinics, Singapore, Singapore

**Keywords:** Multimorbidity, Physical activity, Health-related quality of life, Asian, Primary care, Cross-sectional

## Abstract

**Background:**

The co-occurrence of two or more chronic medical conditions in an individual is defined as multimorbidity. Lifestyle factors, including poor dietary patterns, physical inactivity, tobacco use, and excessive alcohol consumption are key modifiable risk factors that play a role in the development of chronic medical conditions and potentially multimorbidity. The current study aimed to examine the level of physical activity among those with multimorbidity and its association with socio-demographic factors, clinical parameters, and health-related quality of life (HRQoL) among community-dwelling adults attending a primary care clinic in Singapore.

**Methods:**

This cross-sectional study was conducted among patients with multimorbidity between August 2014 and June 2016. Physical activity was measured using the International Physical Activity Questionnaire (IPAQ) Short Form. HRQoL was measured using the EuroQol-5 Dimension (EQ-5D-3 L). Data on clinical parameters including hemoglobin A1c (HbA1C), low-density lipoprotein cholesterol (LDL-C), and blood pressure were collected from patient records. Multivariable logistic regression analysis and linear regression were performed to determine the association between IPAQ and clinical health outcomes, as well as HRQoL measures, respectively.

**Results:**

In all, 932 respondents with multimorbidity were recruited for the study. Of these, 500 (53.8%) had low physical activity, 325 (35.0%) had moderate physical activity, while 104 (11.2%) had high physical activity. Respondents who were insufficiently active had significantly higher odds of being overweight/ obese (OR: 1.5, 95% confidence interval [CI]: 1.1–1.9, *p* = 0.01) as compared to those who were sufficiently physically active. The multiple linear regression model revealed that insufficient activity level was negatively associated with EQ-5D index score (β = − 0.05, *p* <  0.001) and the visual analogue scale (β = − 4.4, *p* <  0.001) measuring HRQoL as compared to sufficient activity levels in respondents with multimorbidity.

**Conclusions:**

The low levels of physical activity among patients with multimorbidity, and its association with overweight status and poorer HRQoL emphasizes the importance of increasing physical activity in this population. Family physicians treating patients with chronic diseases need to continue encouraging and helping individuals to initiate and maintain appropriate physical activity levels.

## Introduction

The co-occurrence of multiple chronic medical conditions in an individual has been defined as multimorbidity in the research literature [1, 2]. Boyd and Fortin [3] defined multimorbidity more precisely as “the co-existence of two or more chronic conditions (physical and mental) in the same individual, where one condition is not necessarily more central than the other” [3]. A meta-analysis of 39 studies which included primary care patients in 12 countries suggested that the prevalence of multimorbidity was highly variable and dependent upon the population under consideration, varying from a low of about 13% in those aged 18 years and above to 95% in those aged 65 years and older [4]. Multimorbidity is associated with several adverse outcomes which include psychological stress [5, 6], a lower quality of life [7, 8], appointments with multiple health care providers for their different conditions, polypharmacy and multiple behavioral recommendations [9–11], and increased use of health care services and resultant costs [12, 13], thus presenting a significant challenge to both patients and health care providers everywhere [14].

Singapore is a Southeast Asian multi-ethnic nation with a resident population of about 4.0 million, of which the majority identify as being of Chinese ethnicity (about 70%), followed by those of Malay, and Indian ethnicity [15]. The prevalence of multimorbidity is increasing in Singapore as elsewhere, mainly due to the increasing life expectancy in tandem with improvements in the provision of medical care and public health interventions [16, 17]. 16.3% of Singapore residents in a population survey were found to have two or more chronic medical conditions with hypertension or high blood pressure being reported most frequently (20%), followed by diabetes or high blood sugar (9%). Among respondents with any two chronic conditions, the most common combinations were “hypertension/high blood pressure and high blood sugar/diabetes” (23.9%) (18). The study also found that health-related quality of life (HRQoL) among those with multimorbidity was found to be significantly lower as compared to those without chronic conditions [18]. A study among older adults attending primary care clinics in Singapore similarly found that the most commonly reported chronic conditions were hypertension, hyperlipidemia, and diabetes, and multimorbidity was associated with lower HRQoL [19].

While population ageing is associated with multimorbidity, several other socio-demographic factors that play a role the growing prevalence of multimorbidity must be acknowledged. Lifestyle factors such as poor dietary patterns, physical inactivity, tobacco use, and excessive alcohol consumption are other key proximal factors that play a role in the pathogenesis of chronic medical conditions [20, 21]. Regular participation in physical activity is found to be effective in the primary and secondary prevention of several chronic diseases, including cardiovascular, metabolic, psychiatric, and neurological diseases [22, 23]. Relative to individuals with insufficient physical activity, physically active males and females show lower rates of all-cause as well as cause-specific mortality [24–28]. Research studies examining the association between multimorbidity and physical activity have demonstrated equivocal results, with some studies finding an association [29, 30] while others did not show any association between physical activity and multimorbidity [31, 32].

Physical activity contributes to multiple domains of quality of life. Using an open-ended questionnaire, Gill et al., found that physical activity not only contributed to the physical but also to the social and spiritual domains of quality of life [33]. Studies in the general population have shown a consistently positive association between self-reported physical activity and HRQoL [34]. Thus, not surprisingly, physical activity also improves HRQoL in patients with chronic medical conditions like cardiovascular disease [35], diabetes [36], and stroke [37]. However, the effect of physical activity on HRQoL amongst patients with multimorbidity is not well studied.

Thus, the current study aimed to examine the levels of physical activity among those with multimorbidity and its association with socio-demographic factors, clinical parameters, and HRQoL among community-dwelling adults attending a primary care clinic in Singapore. We hypothesised that majority of patients with multimorbidity would have low physical activity and that low physical activity would be associated with poorer control of the chronic physical conditions and a lower quality of life.

## Methods

### Sample

This cross-sectional study was conducted between August 2014 and June 2016 in a primary care clinic which is part of the National Healthcare Group (NHG), serving the northern part of Singapore with an average daily attendance of about 1400 patients. The primary care clinics referred to as ‘polyclinics’ in Singapore provide a comprehensive range of health services, such as providing treatment for acute medical conditions, management of chronic diseases, women and child health services, and dental care. All patients who were: (i) aged 21 years and above (ii) diagnosed with current co-existence of three most prevalent chronic conditions, i.e., hyperlipidaemia, hypertension, and diabetes mellitus Type 1 or 2 (i.e., diagnosed with all three conditions) (iii) able to understand spoken English, Mandarin, Malay or Tamil and (iv) seen at the Polyclinic at least twice in the 6 months prior to recruitment were included in the study.

The current study was part of a larger study examining multimorbidity in a primary care setting. To ensure the achievement of the individual aims of the study, various sample sizes were calculated. The largest sample size was used to ensure that the study had enough power to answer all the research questions. Taking into account 5% missing data whereby listwise deletion could be safely practiced, a sample size of 892 was considered desirable. Further, assuming a 50% response rate from the patients approached for the study, a sample size of 1800 was considered reasonable. A random sample of 1800 patients who met the inclusion criteria was drawn from the patient population and tagged using the clinic list. The sample was released in 4 replicates as only one research assistant worked full time on the project, and this ensured a good outreach. In all, 1366 patients were approached of whom 932 patients agreed to participate in the study – resulting in an acceptable response rate of 68.2%.

Clinicians and front-line staff referred the patients to the research assistant. Potential participants from the sample were approached before/during/after their scheduled appointments (i.e., regular follow up for their chronic conditions) at the Polyclinic and invited to participate in the study. Participants included were clinically stable (not acutely ill) and determined to be cognitively capable of providing informed consent and participating in the research which took about 30 min. Trained research assistants conducted the interviews in the language preferred by the respondent. The study questionnaire was programmed on the QuickTapSurvey (www.quicktapsurvey.com) app on a tablet computer. On completion of the study respondents were paid SGD 30 as inconvenience fee. The Domain Specific Review Board, National Healthcare Group, Singapore (Ethics Committee) approved the conduct of the study, and all respondents provided written informed consent before participating in the study.

### Questionnaires

#### International Physical Activity Questionnaire Short Form (IPAQ-SF)

The 7-item IPAQ- SF questionnaire assesses a person’s physical activity undertaken as part of their daily life [38]. The first six questions of IPAQ-SF ask about three specific types of activity in the last seven days, namely: walking, moderate-intensity activities, and vigorous-intensity activities. Respondents are then asked about the specific number of days and amount of time in minutes which they spend doing these respective activities. The last question deals with the amount of time ‘spent sitting’ on workdays. Using the criteria provided in the IPAQ scoring protocol [39], daily and weekly metabolic equivalents of a task (MET) values were calculated using the same formulas recommended as follows:

$$ {\displaystyle \begin{array}{c} Walking MET- minutes/ week=\times 3.3\times walking\  minutes\times walking\  days\\ {} Moderate\  MET- minutes/ week=\times 4.0\times moderate- intensity\  activity\  minutes\times moderate\  days\\ {} Vigorous\  MET- minutes/ week=\times 8.0\times vigorous- intensity\  activity\  minutes\times vigorous- intensity\  days\\ {}\mathrm{Total}\ \mathrm{PA}\ \mathrm{MET}-\mathrm{minutes}/\mathrm{week}=\mathrm{sum}\ \mathrm{of}\ \mathrm{walking}+\mathrm{moderate}+\mathrm{vigorous}\ \mathrm{MET}-\mathrm{minutes}/\mathrm{week}\ \mathrm{scores}.\end{array}} $$

Three levels of physical activity have been proposed by the IPAQ group [39]:

- Low: Those not meeting criteria for either moderate or high physical activity as defined below were categorized in this group.
- Moderate: This includes achieving a minimum of at least 600 MET-min/week OR 3 or more days of vigorous activity of at least 20 min per day OR 5 or more days of moderate-intensity activity OR walking of at least 30 min every day in a week.
- High: Comprises achieving a minimum of at least 3000 MET-minutes/week OR vigorous-intensity activity on at least 3 days and accumulating at least 1500 MET-minutes/week.

The American College of Sports Medicine and American Heart Association have recommended (both for adults aged 18–64 years and older adults aged 65 years and above as well as those with chronic physical conditions) moderate intensity aerobic activity for a minimum of 5 days per week or vigorous intensity aerobic activity for a minimum of 3 days per week to promote and maintain health [40]. These values correspond to the ‘moderate’ category of the IPAQ -SF. Thus, for the purposes of this study we have reclassified IPAQ categories into two groups – (i) Low physical activity group as ‘Insufficently active’and (ii) Moderate/High activity group as ‘Sufficently active’ [41].

#### The EuroQol-5 dimension (EQ-5D-3 L)

The EQ-5D-3 L is an instrument that evaluates the generic quality of life and comprises a descriptive system and a Visual Analogue Scale (EQ-VAS) [42]. The descriptive system has five dimensions: mobility, self-care, usual activities, pain/discomfort, and anxiety/depression. Respondents were asked to rate their health on a three-point scale (no problem/moderate problem/extreme problem). The answers given by the respondents result in 243 unique health states and can be converted into a utility score (EQ-5D index) anchored at 0 for death and 1 for perfect health. The utility index used in this study was based on Singapore time trade-off values [43]. The EQ-VAS records the patient’s self-rated health on a vertical visual analogue scale that ranges from 0 for ‘Best imaginable health state’ and 100 for ‘Worst imaginable health state.’ In addition to examining the EQ-5D index and EQ-VAS, the five dimensions of the EQ-5D were also dichotomized (having no problem vs. having moderate/extreme problems) to identify whether respondents had endorsed having issues with said dimensions.

#### Socio-demographic collection form

Demographic information was obtained during the interview. These included the year of birth (age was calculated from the interview date), gender, ethnicity, education level, income and type of housing. These correlates have been found to be associated with physical activity in previous studies [41, 44–47]. The age of the respondents was grouped into four categories – ‘< 55’, ‘55–64’, ‘65–74’, and ‘≥ 75’, ethnicity was classified as Chinese, Malay, Indian and Others. Education level was grouped into four categories – No formal education, Primary, Secondary, and Post-Secondary. Monthly household income was classified into five categories – < SGD 2000, SGD 2000-3999, SGD 4000-5999, SGD 6000-9999, and ≥ 10,000. Type of housing was grouped into four categories – 1/2/3 room Housing Development Board (HDB) (public housing) flats, 4-room HDB flats, 5-room/exceutive HDB/Housing and Urban Development Company (HUDC) and Condominium/Private flats/Landed Property/Private Terrace/Bungalow.

#### Clinical data

Data on hemoglobin A1c (HbA1C; cut off at 7% and above) [48], low density lipoprotein cholesterol (LDL-C; cut off at 2.60 mmol/L and above) [49] and blood pressure (Systolic blood pressure cut off at 140 mm/Hg; Diastolic blood pressure cut off at 90 mmHg) [50] were collected from patient records based on routine clinical monitoring of the patients as a measure of diabetes, dyslipidemia and hypertension control. We obtained the body mass index (BMI) by collecting height and weight of the respondents before the clinic appointment. Based on World Health Organization (WHO) [51] International cut-offs, respondents who had BMI ≥ 25 kg/m^2^ were classified as ‘Overweight/Obese’, while those whose BMI fell within the range of 18.5 to less than 25 were classified as having BMI in the ‘normal range.’ Additionally, we also analysed the BMI based on WHO guidelines for Asian populations, those who had BMI ≥ 23 kg/m^2^ were categorized as ‘Overweight/Obese’, and those in the range of 18.5 to less than 23 were classified to be in the ‘normal range’ [52].

### Statistical analysis

Means and standard deviations were calculated for continuous variables, whereas frequencies and percentages were calculated for categorical variables. A multivariable logistic regression analysis was performed to determine the sociodemographic correlates of physical activity assessed through IPAQ. A series of simple logistic regression analyses were used to determine the association between IPAQ and clinical health outcomes, and the five subscales of the EQ-5D. Simple linear regression analyses were conducted to investigate the association between the IPAQ and EQ-5D index, as well as EQ-VAS scores. Statistical significance was set at the conventional level of *p* <  0.05, using two-sided tests. All statistical analyses were conducted with SPSS version 23.

## Results

In all, 932 respondents with multimorbidity were recruited for the study. Of these, 115 (12.3%) were less than 55 years old, 330 (35.4%) were between 55 and 65, 360 (38.6%) were between 65 and 75, and the remaining 127 (13.6%) were 75 and older. The majority of the sample were Male (55%, *n* = 513). In all 769 patients (82.5%) identified themselves as Chinese, 70 (7.5%) as Malays, 77 as (8.3%) Indians, and the remaining 16 (1.7%) indicated that they were of other ethnicities (e.g., Filipino, Arab, Eurasian, etc.). A cross-tabulation of physical activity levels by socio-demographic and clinical characteristics of the participants is presented in Table 1. Since respondents who fell within the “other” ethnicity category consisted of a diverse set of races and therefore constituted a heterogeneous sample, findings regarding this group are not reported in the present study.

**Table 1:** Cross-tabulation of physical activity levels with sociodemographic characteristics, clinical parameters, EQ-5D Utility Index and EQ-VAS scores of the sample (*n* = 932), and the *p*-values of the sociodemographic variables associated with physical activity in a univariate logistic regression analysis

	Physical Activity Level					*p*-value of variable in a logistic regression analysis		
	Insufficiently Active			Sufficiently Active				
	n	%		n	%	*p*		
Age (in years)								
Less than 55	68	13.6		46	10.7	ref		
55 to less than 65	170	34.0		158	36.8	0.59		
65 to less than 75	187	37.4		173	40.3	0.57		
75 and above	75	15.0		52	12.1	0.72		
Gender								
Male	275	55.0		237	55.2	ref		
Female	225	45.0		192	44.8	0.94		
Ethnicity								
Chinese	423	84.6		345	80.4	ref		
Malay	34	6.8		35	8.2	0.39		
Indian	36	7.2		40	9.3	0.51		
Others	7	1.4		9	2.1	0.49		
Education Level								
No formal education	93	18.6		77	17.9	ref		
Primary/PSLE	164	32.8		129	30.1	0.88		
Secondary/‘N’ Level /‘O’ Level	162	32.4		153	35.7	0.60		
Post-secondary (Diploma/‘A’ Level/Degree/Masters/PhD)	81	16.2		70	16.3	0.70		
Monthly Household Income (SGD)^ab^								
Below $2000	175	48.3		165	50.0	ref		
$2000 - $3999	79	21.8		81	24.5	0.68		
$4000 - $5999	54	14.9		44	13.3	0.62		
$6000 - $9999	31	8.6		28	8.5	0.82		
$10,000 and above	23	6.4		12	3.6	0.15		
Housing Type								
1/2/3 room HDB flat	99	19.8		95	22.2	ref		
4-room HDB flat	217	43.5		170	39.7	0.10		
5-room/Executive HDB flat/HUDC	127	25.5		106	24.8	0.23		
Private Flats/Condominium/Landed Property/Terrace/Bungalow	56	11.2		57	13.3	0.67		
	Physical Activity							
	Insufficiently Active				Sufficiently Active			
	n	%	Mean	S.D.	n	%	Mean	S.D.
HbA1c								
Normal (< 7%)	207	41.5	6.4	0.4	190	44.4	6.3	0.4
High (i.e., ≥ 7% and above)	292	58.5	8.1	1.2	238	55.6	8.1	1.2
LDL-C								
Normal (< 2.60 mmol/L)	398	81.9	2.0	0.4	334	78.4	2.0	0.4
High (i.e., ≥ 2.60 mmol/L)	88	18.1	3.2	0.5	92	21.6	3.2	0.6
Body Mass Index (International Recommendation)								
Normal (≥18.5 & < 25)	160	34.0	22.7	1.5	178	43.0	22.8	1.6
Overweight/Obese (≥ 25)	311	66.0	29.0	3.4	236	57.0	28.9	3.4
Body Mass Index (Asian Recommendation)								
Normal (≥18.5 & < 23)	82	17.4	21.5	1.1	88	21.3	21.43	1.1
Overweight/Obese (≥ 23)	389	82.6	28.0	3.6	326	78.7	27.57	3.6
Blood Pressure								
Normal (i.e. sBP < 140 & dBP < 90)	383	76.8	–	–	335	78.1	–	–
High (i.e. sBP/dBP ≥ 140/90)	116	23.2	–	–	94	21.9	–	–
EQ-5D Mobility								
No problems with mobility	409	81.8	–	–	381	88.8	–	–
Endorses problems with mobility	91	18.2	–	–	48	11.2	–	–
EQ-5D Self-care								
No problems with self-care	482	96.4	–	–	423	98.6	–	–
Endorses problems with self-care	18	3.6	–	–	6	1.4	–	–
EQ-5D Usual activities								
No problems with usual activities	472	94.4	–	–	419	97.7	–	–
Endorses problems with usual activities	28	5.6	–	–	10	2.3	–	–
EQ-5D Pain/Discomfort								
No pain/discomfort	359	71.8	–	–	325	75.8	–	–
Endorses having pain/discomfort	141	28.2	–	–	104	24.2	–	–
EQ-5D Anxiety/Depression								
No anxiety/depression	416	83.2	–	–	383	89.3	–	–
Endorses having anxiety/depresison	84	16.8	–	–	46	10.7	–	–
EQ-5D Index	500	–	0.9	0.2	429	–	0.9	0.1
EQ-VAS	500	–	71.5	15.9	429	–	75.9	14.6

*A Level* Singapore-Cambridge General Certificate of Education Advanced *Level, dBP* Diastolic blood pressure, *EQ-5D* European Quality of Life −5 Dimension, *EQ-VAS* European Quality of Life Visual Analogue Scale, *HbA1C* Haemoglobin A1C, *HDB* Housing Development Board, *HUDC* Housing and Urban Development Company*LDL-C* Low-Density Lipoprotein Cholesterol, *N Level* Singapore-Cambridge General Certificate of Education Normal (Academic) Level, *O Level* General Certificate of Education: Ordinary Level, *PSLE* Primary School Leaving Examination, *sBP* Systolic blood pressure, *SGD* Singapore Dollar

^a^Denotes monthly household income in Singapore dollars in 1 month before participation

^b^238 respondents refused to provide information on their income

### Activity level of sample

Based on the respondents’ self-reports on the IPAQ for seven days preceding the time of the interview, 500 (53.8%) had low physical activity, 325 (35.0%) had moderate physical activity, while 104 (11.2%) had high physical activity. This also indicates that 500 (53.8%) respondents were insufficiently active, while 429 (46.2%) were sufficiently active. Respondents had a mean of a median of 600 MET-minutes/week. The interquartile range of MET-minutes/week was 1440, and ranged from a minimum of 0 to a maximum of 9198.

### Association of IPAQ and socio-demographic variables

A logistic regression analysis consisting of sociodemographic characteristics was conducted to examine their association with physical activity. As displayed in Table 1, results revealed that no sociodemographic variables were significantly associated with physical activity.

### Clinical correlates of physical activity

Five separate logistic regression analyses were conducted to examine whether physical activity was associated with clinical parameters, including HbA1c, LDL-C, blood pressure, and BMI (International and Asian population recommendation). Details of the results are shown in Table 2. The results indicated that respondents who were insufficiently active had significantly higher odds of being overweight/obese based on WHO international recommendation (*p* = 0.01), however, this finding was not replicated with the WHO Asian population recommendation for BMI (*p* = 0.15). Insufficient activity was not significantly associated with HbA1c (*p* = 0.37), LDL-C (*p* = 0.19), or hypertension (*p* = 0.63) among respondents with multimorbidity.

**Table 2:** Logistic regression results of the association between physical activity levels and clinical parameters

	HbA1c^a^			LDL-C^b^			Blood Pressure^c^			BMI – WHO International Guideline^d^			BMI – WHO Asian Guideline^e^		
Variable	OR	95% CI	*p*	OR	95% CI	*p*	OR	95% CI	*p*	OR	95% CI	*p*	OR	95% CI	*p*
Activity Level															
Insufficiently Active	1.1	0.9–1.5	0.37	0.8	0.6–1.1	0.19	1.1	0.8–1.5	0.63	**1.5**	1.1–1.9	**0.01**	1.3	0.9–1.8	0.15
Sufficiently Active	ref			ref			ref			ref			ref		

Bold print highlights statistically significant odds ratio

*BMI* Body mass index, *CI* Confidence interval of Odds Ratio, *HbA1C* Haemoglobin A1C, *LDL-C* Low-density lipoprotein Cholesterol, *OR* Odds ratio, *WHO* World Health Organization

^a^High HbA1c (i.e., equal to 7% and above) *n* = 531 (57.1%), normal HbA1c (reference group) *n* = 399 (42.9%);

^b^High LDL-C (i.e., equal to 2.60 mmol/L and above) *n =* 181 (19.8%), normal LDL-C (reference group) *n* = 734 (80.2%);

^c^High blood pressure (i.e., equal to or above 140/90) *n* = 211 (22.6%), normal blood pressure (reference group) *n* = 720 (77.3%);

^d^Overweight/Obese (i.e. ≥ 25 kg/m^2^) *n* = 550 (61.9%), normal range (≥ 18.5 & < 25) (reference group) *n* = 338 (38.1%);

^e^Overweight/Obese (i.e. ≥ 23 kg/m^2^) *n* = 718 (80.9%), normal range (≥ 18.5 & < 23) (reference group) *n* = 170 (19.1%)

### Physical activity and health-related quality of life (HRQoL)

Two linear regression analyses were conducted to examine whether physical activity was significantly associated with HRQoL as measured by the EQ-5D utiliy index and EQ-VAS. Results of the linear regression analyses are presented in Table 3. Result indicated that being insufficient active was associated with lower EQ-5D index scores (β = − 0.05, 95% CI: − 0.08 - -0.03, *p* <  0.001) as compared to being sufficiently active in respondents with multimorbidity. Similarly, results also indicated that insufficient activity was significantly associated with lower scores on the EQ-VAS (β = − 4.4, 95% CI: − 6.4 - -2.4, *p* < 0.001).

**Table 3:** Linear regression results of physical activity levels associated with Health-Related Quality of Life (EQ-5D index and EQ-VAS)

	EQ-5D Index			EQ-VAS		
Variable	β	95% CI	*p*	β	95% CI	*p*
Activity Level						
Insufficiently Active	**−0.05**	**−0.08 - -0.03**	**< 0.001**	**−4.4**	**−6.4 - -2.4**	**< 0.001**
Sufficiently Active	ref			ref		

Bold print highlights statistically significant β value

*EQ-5D* European Quality of Life - 5 Dimension, *β* standardized coefficient, *95% CI* 95% confidence interval of β

Five separate logistic regression analyses were conducted to examine the association of physical activity levels with each of the five subscales of the EQ-5D. Results of the logistic regression analyses are presented in Table 4. Results indicated that respondents who were insufficiently active were significantly more likely to endorse having problems with mobility (OR: 1.8, 95% CI: 1.2–2.6, *p* = 0.003), self-care (OR: 2.6, 95% CI: 1.0–6.7, *p* = 0.04), usual activities (OR: 2.5, 95% CI: 1.2–5.2, *p* = 0.02), and anxiety and depression (OR: 1.7, 95% CI: 1.1–2.5, *p* = 0.01) as compared to those who were sufficiently active. In contrast, there was no significant association between physical activity and endorsement of problems with pain and discomfort (*p* = 0.17).

**Table 4:** Logistic regression results of physical activity levels associated with EQ-5D subscales

	Mobility^a^			Self-care^b^			Usual Activities^c^			Pain /Discomfort^d^			Anxiety / Depression^e^		
Variable	OR	95% CI	*p*	OR	95% CI	*p*	OR	95% CI	*p*	OR	95% CI	*p*	OR	95% CI	*p*
Activity Level															
Insufficiently Active	**1.8**	1.2–2.6	**0.003**	**2.6**	1.0–6.7	**0.04**	**2.5**	1.2–5.2	**0.02**	1.2	0.9–1.7	0.17	**1.7**	1.1–2.5	**0.01**
Sufficiently Active	ref			ref			ref			ref			ref		

Bold print highlights statistically significant odds ratio

*EQ-5D* European Quality of Life - 5 Dimension, *OR* odds ratio, *95% CI* 95% confidence interval of odds ratio

^a^endorses problems with mobility *n* = 139 (15.0%), no problems with mobility (reference group) *n* = 790 (85.0%);

^b^endorses problems with self-care *n =* 24 (2.6%), no problems with self-care (reference group) *n* = 905 (97.4%);

^c^endorses problems with usual activities *n* = 38 (4.1%%), no problems with usual activities (reference group) *n* = 891 (95.9%);

^d^endorses having pain/discomfort *n* = 245 (26.5%), no pain/discomfort (reference group) *n* = 684 (73.5%);

^e^endorses anxiety/depression *n* = 130 (14.1%), no anxiety/depression (reference group) *n* = 799 (85.9%)

## Discussion

The current study among a multi-ethnic population of patients with multimorbidity found that the majority had low physical activity (53.8%). Thus, in line with our hypothesis, the majority of people with multimorbidity do not meet the recommended standards of physical activity that ensures maintenance of optimal health. The prevalence of low physical activity was more than that observed in the general population of Singapore. Using data from 4302 adults assessed with the Global Physical Activity Questionnaire and using the same cut-off as that adopted by the current study, the Singapore National Health Survey 2010 (NHS) found that the prevalence of those with inactive levels (less than a moderate level of physical activity) was 37.4% [53]. Another local study among 4750 community-dwelling adults that used a questionnaire adapted from several established and validated questionnaires to assess physical activity found that 71% of those who participated in the study achieved the recommended level of activity of at least 150 min per week of moderately intense physical activity or 60 min of vigorous physical activity or a combination of both [54]. This meant that 29% of the participants did not achieve the recommended criteria of at least 150 min per week of physical activity; this group corresponds to those with low activity in our study. The Irish Longitudinal Study on Ageing assessed 8175 community-dwelling residents aged 50 years and older using the IPAQ and categorized them as low, moderate and high activity based on the same criteria as the current study [55]. The prevalence of low activity among those with multimorbidity in the Irish study was 30% which was lower than that found in the current study.

Those with low physical activity levels were more likely to be overweight/ obese as per the WHO International guidelines [51]. Other studies have found that physical inactivity was associated with both obesity and multimorbidity [56, 57], and being overweight or obese was an independent risk factor for multimorbidity [58, 59]. While the cross-sectional nature of the study prevents us from drawing causal inferences, tackling physical inactivity and obesity among these patients does hold some promise in preventing the accrual of other conditions and further worsening of multimorbidity over time [55]. There is evidence to suggest that even small improvements in physical activity can result in clinically relevant reduction in the overall risk of chronic conditions like cardiovascular diseases as well as all-cause mortality [60, 61].

Those with low levels of physical activity level were associated with a poorer quality of life, especially in mobility, self-care, usual activities and anxiety and depression domains. Using structural equation modeling, Maddigan et al. [62] observed a positive relationship between exercise adherence and HRQoL. The authors also found that higher BMI was associated with worse HRQoL, while exercise adherence was associated with lower BMI, thereby suggesting a direct or indirect effect of physical activity on HRQoL. Domains such as self-care and usual activities which were negatively associated with low physical activity affect independence and functioning and a lower physical activity level may thus contribute to a higher risk of disability in this group of patients with multimorbidity. It is also possible that other factors, e.g., greater disease burden, depressive symptoms, and financial constraints may have contributed to both lower HRQoL and lower physical activity in this population [63].However, we did not examine disease severity or depressive symptoms in the current study. In line with results of previous studies [64, 65], low physical activity was also associated with lower EQ-VAS scores in the current study. Studies suggest that the EQ-VAS measures the respondent’s overall rating of their health which is a broader underlying construct than that measured by EQ-5D and represents a global health rating from the individual’s perspective [66]. Staying sufficiently active may thus have a significant positive effect on the overall wellbeing of the person.

## Limitations

A considerable strength of the study was the random selection of patients with multimorbidity. However, only about 68% of those whom we approached agreed to participate in the study - thus limiting the generalizability of our findings to some extent. Exploring the differences between those who participated and those who did not found that the gender composition of the two groups was statistically significant (*p* = 0.017). There were significantly more men than women in the study as more women than men declined to participate. A significant proportion of those who participated in the study (*n* = 238) refused to provide information on their monthly income leading to respondents with missing data in this category. Socio-economic correlates may have an important association with physical activity which may have been missed in the current study as a result of the missing data. The cross-sectional design did not allow us to determine causation, and we cannot ignore the potential of reverse causation between physical activity and BMI as well as quality of life. The current study only focused on examining the prevalence and effect of physical activity on multimorbidity, though multiple factors are determined to be equally important in other studies [67]. Thus, these lifestyle factors must also be examined in future studies. While the IPAQ-SF cutoff for moderate category identifies those meeting aerobic activity levels suggested by the American American College of Sports Medicine and American Heart Association guidelines, these may not be applicable globally. Finally, while the IPAQ is a valid and widely used measure of physical activity, the limitations of self-reported health behaviours are well-established. Garriguet and Colley [68] found that differences between self-reported physical activity and accelerometer-measured moderate-to-vigorous physical activity could be as much as 37.5 min in either direction.

## Conclusion

The current study is among the first in Singapore that adds to the understanding of the association between multimorbidity and physical activity. In line with our hypothesis, majority of patients with multimorbidity had low physical activity which was associated with poorer HRQoL. Our findings add to the literature emphasizing the importance of increasing physical activity among people with chronic conditions and those with multimorbidity given the benefits of physical activity [60, 61]. Further research is needed to understand populations at greatest risk, the temporality of relationship between physical activity and multimorbidity, appropriate interventions and their mode of delivery which is acceptable to the local population, and most importantly, the health outcomes associated with these interventions in the population over time. Meanwhile, family physicians treating patients with chronic diseases need to continue encouraging and helping individuals to initiate and maintain appropriate physical activity levels.

## Data Availability

The datasets used and/or analysed during the current study are available from the corresponding author on reasonable request.

## Abbreviations

EQ-5D-3 L: EuroQol-5 Dimension
HbA1C: Hemoglobin A1c
HRQoL: Health-Related Quality of Life
IPAQ-: International Physical Activity Questionnaire
LDL-C: Low-density lipoprotein cholesterol
MET: Metabolic equivalent for task
NHG: National Healthcare Group
VAS: Visual Analogue Scale
WHO: World Health Organisation
